# Optimal treatment strategy for patients with pancreatic cancer having positive peritoneal cytology: A nationwide multicenter retrospective cohort study supervised by the Japanese Society of Hepato‐Biliary‐Pancreatic Surgery

**DOI:** 10.1002/jhbp.12074

**Published:** 2024-09-24

**Authors:** Kyohei Ariake, Masamichi Mizuma, Michiaki Unno, Sohei Satoi, Naoto Yamamoto, Masamichi Hayashi, Manabu Kawai, Hirofumi Akita, Eiji Toyoda, Tsutomu Fujii, Masaru Sasaki, Kenichi Hakamada, Jota Watanabe, Etsuro Hatano, Masaaki Hidaka, Satoshi Hirano, Hiroshi Kurahara, Ippei Matsumoto, Goro Honda, Toshiro Ogura, Masafumi Nakamura, Itaru Endo

**Affiliations:** ^1^ Department of Surgery Tohoku University Graduate School of Medicine Sendai Japan; ^2^ Department of Gastroenterological Surgery Sendai City Medical Center Sendai Open Hospital Sendai Japan; ^3^ Department of Surgery Kansai Medical University Hirakata Japan; ^4^ Department of Gastrointestinal Surgery Kanagawa Cancer Center Yokohama Japan; ^5^ Department of Surgery, Graduate School of Medicine Nagoya University Nagoya Japan; ^6^ Second Department of Surgery Wakayama Medical University School of Medicine Wakayama Japan; ^7^ Department of Gastroenterological Surgery Osaka International Cancer Institute Osaka Japan; ^8^ Department of Surgery Otsu Red Cross Hospital Otsu Japan; ^9^ Department of Surgery and Science, Faculty of Medicine Academic Assembly, University of Toyama Toyama Japan; ^10^ Department of Surgery JA Hiroshima General Hospital Hatsukaichi Japan; ^11^ Department of Gastroenterological Surgery Hirosaki University Graduate School of Medicine Hirosaki Japan; ^12^ Department of Gastrointestinal Surgery Ehime Prefectural Central Hospital Matsuyama Japan; ^13^ Department of Surgery Graduate School of Medicine, Kyoto University Kyoto Japan; ^14^ Department of Digestive and General Surgery Shimane University Faculty of Medicine Izumo Japan; ^15^ Department of Gastroenterological Surgery II Hokkaido University Faculty of Medicine Sapporo Japan; ^16^ Department of Digestive Surgery Kagoshima University Kagoshima Japan; ^17^ Department of Surgery, Faculty of Medicine Kindai University Osakasayama Japan; ^18^ Department of Surgery Institute of Gastroenterology, Tokyo Women's Medical University Tokyo Japan; ^19^ Department of Gastroenterological Surgery Saitama Cancer Center Saitama Japan; ^20^ Department of Surgery and Oncology Graduate School of Medical Sciences, Kyushu University Fukuoka Japan; ^21^ Japanese Society of Hepato‐Biliary‐Pancreatic Surgery Tokyo Japan; ^22^ Department of Gastroenterological Surgery, Graduate School of Medicine Yokohama City University Yokohama Japan

**Keywords:** chemotherapy, conversion surgery, pancreatic cancer, peritoneal cytology

## Abstract

**Background:**

The aim of this study was to determine the optimal treatment for patients with pancreatic cancer (PaCa) having positive peritoneal cytology (PPC).

**Methods:**

This multicenter retrospective study included patients with PPC treated at 78 high‐volume centers between January 2012 and December 2020. Prognoses after resection (S‐group) and initiation of nonsurgical treatment (N‐group) were compared. Prognostic factors for survival in both groups were analyzed. Detailed characteristics of conversion surgery (CS) in the N‐group were evaluated.

**Results:**

In total, 568 enrolled patients were classified into an S‐group (*n* = 445) or an N‐group (*n* = 123). Median survival times (MSTs) were 19.0 months and 19.3 months, respectively, with no significant difference in prognosis (*p* = .845). The intervenable prognostic factors for survival were adjuvant treatment in the S‐group (*p* < .001) and CS in the N‐group (*p* < .001). Following CS, the MST was prolonged to 45.6 months, and peritoneal or liver recurrence decreased considerably. CS can be expected if PPC is diagnosed before neoadjuvant treatment and when combination treatment is initiated.

**Conclusion:**

Surgical resection may not be beneficial for improving survival when PPC is evident. Chemotherapy aiming for CS may be the optimal treatment for such patients.

## Graphical abstract

In this nationwide retrospective study, Ariake and colleagues revealed that surgical resection may not improve survival in pancreatic cancer patients with positive peritoneal cytology. Conversion surgery is expected for approximately 25% of patients and markedly improves the outcomes, suggesting that chemotherapy aiming for conversion surgery is optimal for such patients.

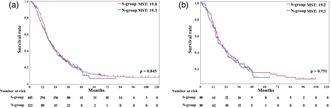

## 1 INTRODUCTION

In patients with pancreatic cancer (PaCa), surgical resection remains the sole curative option. However, postoperative recurrence remains frequent,^1^, ^2^ and surgical indications should be carefully considered. Positive peritoneal cytology (PPC) is classified as distant metastasis (M1) by the American Joint Committee on Cancer Staging Manual (eighth edition),^3^ and the Union for International Cancer Control (UICC) TNM classification (eighth edition).^4^ In Japan, the classification of PaCa also revised the PPC from M0 to M1 in 2023.^5^ On the basis of these classifications, surgical resection for PPC is not recommended by the National Comprehensive Cancer Network (NCCN),^6^ or the latest clinical practice guidelines in Japan.^7^, ^8^ However, these recommendations were based on studies reporting a poor prognosis involving a median survival time (MST) of 20 months for patients with PPC,^9^, ^10^, ^11^, ^12^ and surgical resection was not rejected because of the inferiority of a nonsurgical approach. Therefore, the appropriateness of avoiding surgical resection for PPC remains to be assessed.

There is limited evidence supporting nonsurgical treatment for PPC. To date, few studies have evaluated the prognosis of patients with PPC following nonsurgical treatment. One study reported that the MST of nonsurgical treatment was 13.0 months when patients with locally advanced cancer were included and conversion surgery (CS) was not performed.^13^ Another study reported that initiating systemic chemotherapy and adding CS to selected patients resulted in a favorable prognosis, with an MST of 31.4 months.^14^ These studies suggested that continuation of only chemotherapy is insufficient for treating PPC and that preceding chemotherapy followed by CS might be an effective approach.

PPC is classified as an M1 disease; however, further evidence is needed to establish the optimal treatment, including the benefits of surgical resection. Accordingly, the Japanese Society of Hepato‐Biliary‐Pancreatic Surgery (JSHBPS) conducted a multicenter collaborative project. As the world's largest study on PPC to date, its goal was to determine the optimal treatment for patients with PaCa having PPC concerning resection or nonsurgical treatment, including chemotherapy intended for CS.

## 2 METHODS

### 2.1 Eligibility criteria

This study included patients with PaCa having PPC who were treated between January 2012 and December 2020 at advanced‐level training institutes registered with the JSHBPS committee.^15^ PPC was defined as class IV or V according to the Papanicolaou classification.^16^ The exclusion criteria were as follows: active concomitant cancer; class I, II, III, or unclear cytological status; underwent pancreatectomy other than pancreatoduodenectomy, distal pancreatectomy, or total pancreatectomy; and distant metastasis (i.e., peritoneal dissemination) or avoidance of surgical resection owing to locally advanced factors. Among the eligible patients, those who underwent surgical resection for PPC were assigned to the surgery group (S‐group), whereas those who received nonsurgical treatment were assigned to the nonsurgical treatment group (N‐group). Following nonsurgical treatment, CS was performed for patients who achieved a negative conversion of the cytological status and had no other evident unresectable factors. These patients were included in the N‐group. A detailed group classification of the treatment patterns is summarized in Figure S1–S7.

### 2.2 Study design

This nationwide multicenter retrospective cohort study involved 78 high‐volume centers in Japan (Table S1–S3). The primary endpoint was overall survival (OS) following both surgical resection and nonsurgical treatment for patients with PaCa having PPC. The secondary endpoints were the prognostic factors following surgical resection and the initiation of nonsurgical treatment. Additionally, this study analyzed the number of patients who achieved CS and evaluated the factors that predict CS achievement.

### 2.3 Ethical considerations

This study was approved by the Institutional Review Board Committee of Tohoku University (Sendai, Japan) as a supervising center on November 24, 2022 (2022–1‐730). Approval was obtained from the review board committee at each center. This study was conducted in accordance with the principles of the Declaration of Helsinki. The requirement for informed consent was waived owing to the study's retrospective design. This study was registered with the University Hospital Medical Information Network Clinical Trials Registry (http://www.umin.ac.jp/ctr) (registration no.: UMIN000048808).

### 2.4 Data collection and evaluation

First, a primary survey was performed to investigate the number of patients with PPC and the treatment strategies at each institute. In a secondary survey, treatment policies concerning staging laparoscopy and intraoperative diagnosis of peritoneal cytology were investigated. Using a questionnaire in Excel, clinical and pathological information regarding PPC was collected from medical records, surgical records, and pathological reports maintained at each center. Data collection began on December 12, 2022 and was completed on August 26, 2023. The following clinicopathological factors were analyzed: patient demographics/background (age, sex, tumor location, tumor size, resectability classification based on the NCCN guidelines,^6^ serum carbohydrate antigen 19–9 [CA19‐9] level at PPC diagnosis, cytology classification, and timing of PPC diagnosis before treatment or after neoadjuvant treatment [NAT]); nonsurgical treatment after a PPC diagnosis (treatment regimens, duration, cytology status, and CS achievement); operative factors (operative procedure, blood loss, operative time, and combined vascular resection); pathological factors (tumor size, anterior serosal invasion, retroperitoneal invasion, portal vein invasion, arterial invasion, T and N status according to the UICC TNM classification [eighth edition],^4^ differentiation, and residual tumor classification); and postoperative factors (postoperative complications, pancreatic fistula defined by the International Study Group of Pancreatic Fistula,^17^ in‐hospital death, postoperative serum CA19‐9 level, and adjuvant treatment regimens and durations). CA19‐9 levels ≤37.0 U/mL were considered normal. Patients with CA19‐9 levels ≤2.0 U/mL were considered nonsecretors of CA19‐9^18^ and were excluded from further analyses involving CA19‐9. If the CA19‐9 level was evaluated at a serum bilirubin level >3.0 mg/dL, the patient was excluded from further analysis.

### 2.5 Cytological examination method

Following laparotomy or laparoscopic exploration, peritoneal lavage and cytological examination were conducted by instilling 20–100 mL of warm saline into the abdominal cavity within the patient's pelvis. The maximum fluid volume was obtained after gentle agitation. Smears were generated from centrifuged deposits and examined by pathologists using conventional Papanicolaou or Giemsa staining. Outcomes were acquired intraoperatively (rapid diagnosis) or postoperatively (permanent diagnosis).

### 2.6 Statistical analysis

Statistical analyses were performed using JMPproVer16 for Windows (SAS Institute Inc., Cary, NC, USA) software. Quantitative data were analyzed using a Kruskal–Wallis test. Categorical data were analyzed using Pearson's chi‐squared test. Survival rates were calculated using the Kaplan–Meier method. Comparisons between the two groups were performed using log‐rank tests. A logistic regression model was used to identify the predictive factors for CS incidence. A Cox proportional hazards model was used to analyze prognostic factors for OS following surgical resection and nonsurgical treatment. Forest plots of hazard ratios (HRs) for OS were generated for different subgroup analyses. OS was calculated from the date of PPC diagnosis to the date of death or censoring. To overcome biases owing to the different distributions of covariates among patients who underwent surgical resection and nonsurgical treatment, propensity score matching analysis was performed. A model was used to obtain a one‐to‐one match using the nearest‐neighbor matching method with a caliper of 0.2. The matching algorithm was based on logistic regression without replacement, until all possible matches were formed. The following covariates were matched for age, sex, tumor location, resectability classification, tumor size, CA19‐9 level (at the time of PPC diagnosis), cytology class, and timing of PPC diagnosis. A *p*‐value < .05 indicated statistical significance.

## 3 RESULTS

### 3.1 Current treatment strategy in Japan for patients with PaCa having PPC

The results of the primary survey across 86 centers indicated that the treatment policy for PPC was surgical resection at 52 centers and nonsurgical treatment at 32 centers. In two centers, the treatment policy differed depending on the surgical procedure. The detailed treatment strategies, including the secondary survey, are summarized in Table S2.

### 3.2 Study cohort and patient characteristics

**FIGURE 1:**
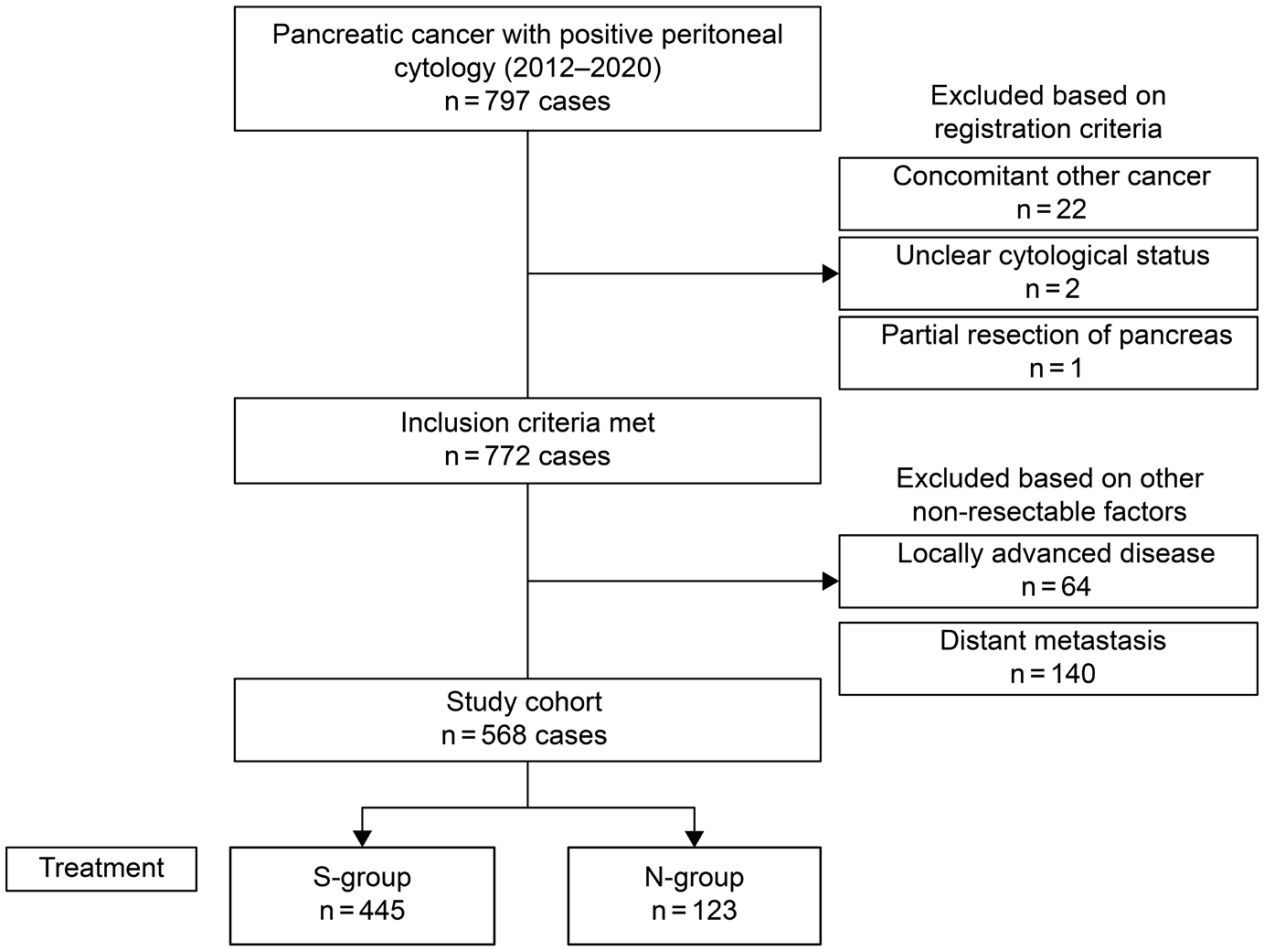
Flow chart of patients included in the study cohort.

In total, 797 patients with PPC were registered at 78 centers. Of these, 25 patients were excluded based on the registration criteria. Moreover, 204 patients were excluded owing to other unresectable factors (64 with locally advanced disease, 140 with distant metastases to other sites). A total of 568 patients were enrolled in this study (Figure 1); 445 patients in the S‐group underwent resection for PPC, whereas 123 patients in the N‐group underwent nonsurgical treatment. The clinicopathological characteristics are summarized in Table 1.

**TABLE 1:** Clinical characteristics of patients in the unmatched and matched cohorts.

Factor		Before matching (Total cohort)			After matching		
		Surgical resection (*n* = 445)	Nonsurgical treatment (*n* = 123)	*p*‐value	Surgical resection (*n* = 89)	Nonsurgical treatment (*n* = 89)	*p*‐value
Age (years)	Median (range)	70 (36–91)	70 (35–88)	.682	73 (40–90)	72 (49–88)	.882
Sex	Male/female	238/207	67/56	.846	45/44	50/39	.453
Tumor location	Head/body‐tail/entire	182/254/9	61/61/1	.176	42/47/0	42/46/1	.603
Resectability classification	Resectable/borderline resectable	353/92	82/41	.003	67/22	66/23	.863
Tumor size (mm)	Median (range)	30 (9–115)	29 (0–70)	.042	25.1 (10–70)	27 (0–70)	.700
Pretreatment CA19‐9 level (U/mL)	Median (range)	171 (0–42 679)	144 (2.8–15 223)	.122	147 (3–8348.7)	153.4 (2.8–15 223)	.666
Cytology class	Class IV/class V	61/384	5/118	.003	7/82	5/84	.550
Timing of PPC diagnosis	After NAT/before NAT	112/333	38/85	.202	16/73	19/70	.572

Abbreviations: CA19‐9, carbohydrate antigen; NAT, neoadjuvant treatment; PPC, positive peritoneal cytology.

### 3.3 Prognosis after resection or nonsurgical treatment

**FIGURE 2:**
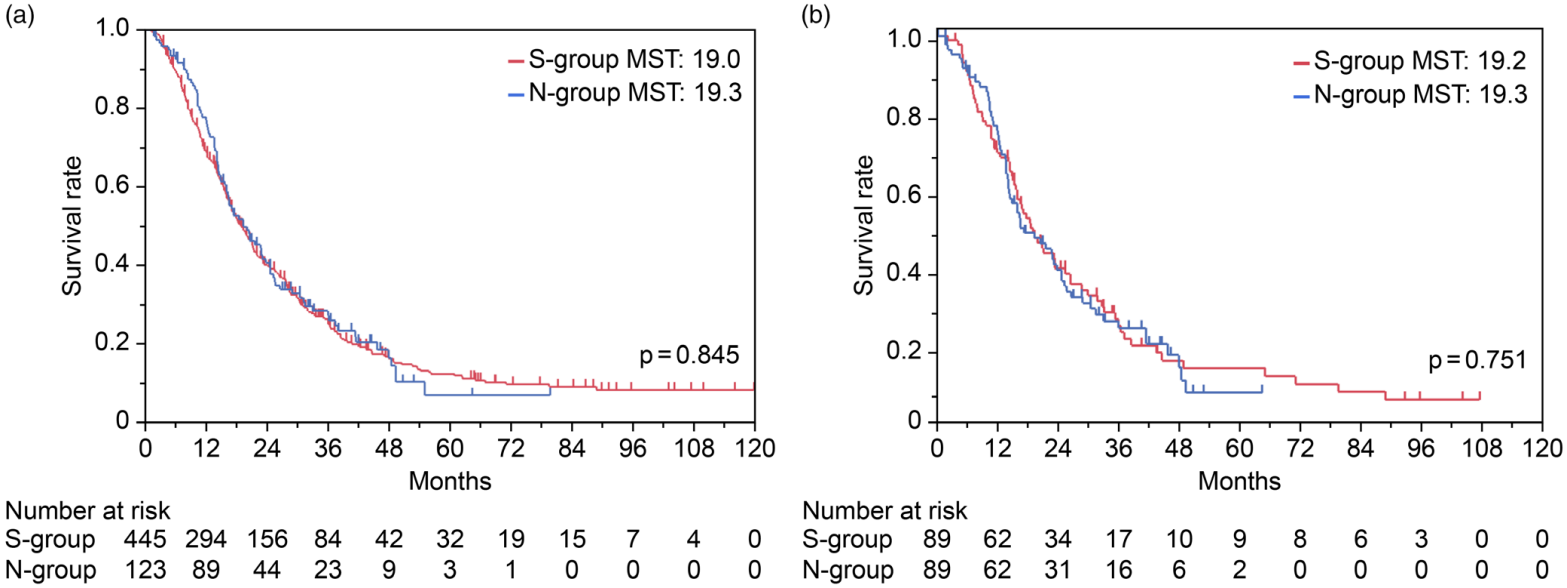
Kaplan–Meier curves of OS. (a) Comparison of OS in patients with PPC in the total cohort. Patients treated with surgical resection (S‐group, *n* = 445) or nonsurgical treatment (N‐group, *n* = 123) are represented in red and blue, respectively. The 1‐, 2‐, 3‐, and 5‐year survival rates were 68.7%, 39.9%, 24.8%, and 12.0%, respectively, in the S‐group and 76.1%, 39.7%, 25.9%, and 6.8%, respectively, in the N‐group. (b) Comparison of OS in patients with PPC after propensity score matching. Patients treated with surgical resection (*n* = 89) or nonsurgical treatment (*n* = 89) are represented in red and blue, respectively. OS was calculated from the date of PPC diagnosis to the date of death or censoring. MST, median survival time; OS, overall survival; PPC, positive peritoneal cytology.

The prognoses did not differ between the S‐group and the N‐group (19.0 months vs. 19.3 months, respectively, *p* = .845; Figure 2a). One‐to‐one propensity score matching (S‐group, 89 patients; N‐group, 89 patients) (Table 1) indicated no significant between‐group difference in prognoses (*p* = .751, Figure 2b). Subgroup analysis was also performed based on various factors using the cohort before matching, but no factors that favored resection over nonsurgical treatment were identified (Figure S2). No significant difference in prognoses was observed between the S‐ and N‐group in patients with resectable PaCa (*p* = .990, Figure S3a) or patients with borderline resectable PaCa (*p* = .237, Figure S3b).

### 3.4 Prognostic factors for OS in the N‐group

Univariable analysis of OS following nonsurgical treatment identified four risk factors: tumor size (*p* = .007), CA19‐9 level at PPC diagnosis (*p* = .004), treatment regimen (*p* = .024), and the presence of CS (*p* < .001). Multivariable analysis of these four factors indicated that CS (hazard ratio [HR]: 5.15, 95% confidence interval [CI]: 2.42–11.0; *p* < .001) and tumor size (HR 2.39, 95% CI: 1.33–4.29; *p* = .004) were prognostic factors (Table 2). The proportion of patients who achieved CS was 24.4%, and the prognosis was extremely favorable; the MST was 45.6 months after PPC diagnosis (Figure S4a) and 33.7 months after CS (Figure S4b). The prognosis after CS was dramatically improved compared with that of patients in the S‐group (*p* < .001; Figure S4a); however, the prognosis of patients with non‐CS was significantly poorer than that of patients in the S‐group (MST, 14.6 months; *p* = .005; Figure S4a).

**TABLE 2:** Prognostic factors for survival in patients with PaCa having PPC after the initiation of nonsurgical treatment.

Factor	Median OS (months)	Univariable analysis		Multivariable analysis	
		HR (95% CI)	*p*‐value	HR (95% CI)	*p*‐value
Age (years)					
≤75 (90)	20.5 (16.2–25.5)				
>75 (33)	16.4 (12.7–22.8)	1.33 (0.84–2.11)	.231		
Sex					
Male (67)	22.6 (16.4–25.5)				
Female (56)	16.2 (12.7–22.8)	1.03 (0.68–1.57)	.872		
Tumor location					
Head (61)	19.3 (14.2–25.2)	1.04 (0.69–1.58)	.838		
Body‐tail (61)	20.5 (15.6–25.6)				
Entire (1)	12.1	3.71 (0.50–27.5)	.200		
Resectability classification					
Resectable (82)	22.6 (16.4–25.2)				
Borderline resectable (41)	16.2 (13.5–20.0)	1.25 (0.81–1.92)	.314		
Tumor size					
≤20 mm (37)	31.4 (20.4–45.6)				
>20 mm (86)	16.2 (14.1–20.5)	1.92 (1.20–3.06)	.007	2.39 (1.33–4.29)	.004
Pretreatment CA19‐9 level					
≤100 U/mL (35)	31.4 (15.9–45.6)				
>100 U/mL (56)	15.9 (12.1–22.6)	2.20 (1.29–3.75)	.004	1.52 (0.85–2.74)	.155
Cytology class					
Class IV (5)	25.2 (22.6–n.r)				
Class V (118)	18.4 (14.8–22.8)	1.51 (0.55–4.13)	.422		
Timing of PPC diagnosis					
After NAT (38)	17.0 (14.1–22.6)	1.23 (0.80–1.90)	.343		
Before NAT (85)	21.5 (15.9–25.5)				
Treatment regimens					
mFOLFIRINOX or GnP (85)	22.8 (17.3–28.8)				
Other or none (38)	15.9 (11.1–17.2)	1.64 (1.07–2.51)	.024	1.58 (0.91–2.73)	.102
Conversion surgery					
Yes (30)	45.6 (31.4–n.r)				
No (93)	14.8 (13.6–17.2)	4.41 (2.48–7.87)	<.001	5.15 (2.42–11.0)	<.001

Abbreviations: CA19‐9, carbohydrate antigen 19–9; CI, confidence interval; GnP, gemcitabine/nab‐paclitaxel; HR, hazard ratio; NAT, neoadjuvant treatment; n.r., not recorded; OS, overall survival; PaCa, pancreatic cancer; PPC, positive peritoneal cytology.

### 3.5 Characteristics and predictive factors for patients with CS compared with non‐CS

Patients with CS had significantly higher rates of resectable PaCa (*p* = .026) and diagnosis before NAT (*p* = .001) compared with non‐CS patients (Table S3). Additionally, CS was performed in all patients after confirming their negative cytological status. The median period from treatment initiation to CS was 6.0 months.

Multivariable analysis for factors contributing to CS identified three predictive factors: CA19‐9 level (≤100 U/mL) at diagnosis (HR 4.62, 95% CI: 1.41–15.2; *p* = .012), a PPC diagnosis before NAT (HR 8.28, 95% CI: 1.50–45.8; *p* = .015), and combination therapy with mFOLFIRINOX or gemcitabine/nab‐paclitaxel (GnP) as the initial treatment (HR 31.9, 95% CI: 3.63–279.2; *p* = .002; Table 3).

**TABLE 3:** Predictive factors for conversion surgery in patients with PaCa having PPC after initiating nonsurgical treatment.

Factor	Conversion rate	Univariable analysis		Multivariable analysis	
		OR (95% CI)	*p*‐value	OR (95% CI)	*p*‐value
Age (years)					
≤75 (90)	28.9%	2.95 (0.94–9.21)	.063		
>75 (33)	12.1%				
Sex (*n*)					
Male (67)	31.3%	2.38 (0.99–5.75)	.053	–	–
Female (56)	16.1%				
Tumor location					
Head (61)/Entire (1)	19.4%			–	–
Body‐Tail (61)	29.5%	1.74 (0.76–4.03)	.192		
Resectability classification					
Resectable (82)	30.5%	3.16 (1.11–9.00)	.031	3.22 (0.82–12.6)	.093
Borderline resectable (41)	12.2%				
Tumor size					
≤20 mm (37)	19.8%	2.20 (0.93–5.19)	.072		
>20 mm (86)	35.1%				
Pretreatment CA19‐9 level					
≤100 U/mL (35)	40.0%	2.73 (1.06–7.01)	.037	4.62 (1.41–15.2)	.012
>100 U/mL (56)	19.6%				
Cytology class					
Class V (118)	23.7%			–	–
Class IV (5)	40.0%	2.14 (0.34–13.5)	.417		
Timing of PPC diagnosis					
After NAT (38)	5.3%			–	–
Before NAT (85)	32.9%	8.84 (1.98–39.4)	.004	8.28 (1.50–45.8)	.015
Initial treatment					
mFOLFIRINOX or GnP (85)	34.1%	19.2 (2.50–146.8)	.005	31.9 (3.63–279.2)	.002
Other (38)	2.6%				

Abbreviations: CA19‐9, carbohydrate antigen 19–9; CI, confidence interval; NAT, neoadjuvant treatment; OR, odds ratio; PaCa, pancreatic cancer; PPC, positive peritoneal cytology.

### 3.6 Patient characteristics and recurrence patterns following CS compared with those in the S‐group

Compared with patients in the S‐group, patients who achieved CS had the following characteristics: a smaller tumor size (*p* < .001), suppressed retroperitoneal invasion (*p* = .004), and less lymph node metastasis (*p* = .032). Moreover, the CS group presented a significantly greater rate of postoperative CA19‐9 normalization than in the S‐group (92.3% vs. 57.9%, respectively; *p* < .001). When the recurrence pattern was analyzed, the rates of peritoneal recurrence (23.3% vs. 47.3%, respectively; *p* = .011) and liver metastasis (10.0% vs. 29.2%, respectively; *p* = .023) were significantly lower in the CS group than in the S‐group (Table 4).

**TABLE 4:** Patient clinicopathological characteristics and recurrence patterns after both surgical resection and conversion surgery.

Factor		Surgical resection (S‐group, *n* = 445)	Conversion surgery (*n* = 30)	*p*‐value
		Total		
Type of operation	PD/DP/TP	176/243/26	12/17/1	.846
Blood loss (mL)	Median (range)	505 (0–9268)	440 (5–4240)	.386
Operation time (min)	Median (range)	361 (106–861)	431 (196–781)	.031
Portal vein resection	Yes (%)	114 (25.6%)	8 (26.7%)	.899
Artery resection	Yes (%)	25 (5.6%)	1 (3.3%)	.594
Comorbidity with Clavien‐Dindo grade 3	Yes (%)	105 (23.6%)	10 (33.3%)	.228
Pancreatic fistula (grade B, C)	Yes (%)	78 (17.7%)	8 (26.7%)	.218
In‐hospital death	Yes (%)	4 (0.9%)	0 (0%)	.602
Tumor size (mm)	Median (range)	33 (0–138)	23.5 (0–48)	<.001
Anterior serosal invasion	Yes (%)	341 (76.8%)	19 (65.5%)	.167
Retroperitoneal invasion	Yes (%)	390 (87.6%)	20 (69.0%)	.004
Portal vein invasion	Yes (%)	163 (36.7%)	9 (31.0%)	.538
Artery invasion	Yes (%)	52 (11.7%)	2 (6.9%)	.430
Pathological T stage classification	Tis/T1/T2/T3/T4	2/29/159/229/24	1/10/8/11/0	<.001
Pathological lymph node metastasis	N0/N1/N2	114/192/139	14/11/5	.032
Differentiation	Well/moderate/poor/other	122/254/47/21	10/13/4/3	.385
Residual cancer	R0/R1	348/97	27/3	.125
Postoperative CA19‐9 level	Median (range)	27.3 (0.4–85817.6)	14.6 (4.1–215)	.004
Postoperative CA19‐9 level	Normal	232 (57.9%)	24 (92.3%)	<0.001
Postoperative treatment	Yes (%)	343 (77.3%)	28 (93.3%)	.039
Regimen of adjuvant treatment	S‐1/GEM/GS/GnP/FOLFIRINOX/other	283/34/10/13/0/3	24/0/0/3/1/0	.002
Duration of adjuvant treatment (month)	≤1/1–3/3–6/>6	19/66/122/132	0/3/12/13	.344
Recurrence site	Peritoneum	207 (47.3%)	7 (23.3%)	.011
	Liver	128 (29.2%)	3 (10.0%)	.023
	Locoregional	65 (14.8%)	6 (20.0%)	.446
	Lung	50 (11.4%)	3 (10.0%)	.813
	Lymph node	39 (8.9%)	4 (13.3%)	.417

Abbreviations: CA19‐9, carbohydrate antigen 19–9; CS, conversion surgery; DP, distal pancreatectomy; GEM, gemcitabine; GnP, gemcitabine/nab‐paclitaxel; NAT, neoadjuvant treatment; PD, pancreaticoduodenectomy; TP, total pancreatectomy.

### 3.7 Prognostic factors for OS in the S‐group

Univariable analysis of OS in the S‐group identified six risk factors: resectability classification (*p* < .001), portal vein resection (*p* = .033), tumor size (*p* = .018), retroperitoneal invasion (*p* = .018), postoperative CA19‐9 level (*p* < .001), and adjuvant treatment (*p* < .001) (Table 5). Multivariable analysis of these six factors indicated that the prognostic factors were borderline resectable PaCa (HR 1.43, 95% CI: 1.07–1.91; *p* = .015), postoperative CA19‐9 non‐normalization (HR 2.04, 95% CI: 1.61–2.58; *p* < .001), and no adjuvant treatment (HR 2.16, 95% CI: 1.63–2.84; *p* < .002). Even when adjuvant treatment was administered in the S‐group, the prognosis was similar to that in the N‐group (*p* = .247) (Figure S5).

**TABLE 5:** Prognostic factors for the survival of patients with PaCa and PPC after surgical resection.

Factor	Median OS (months)	Univariate analysis		Multivariate analysis	
		HR (95% CI)	*p*‐value	HR (95% CI)	*p*‐value
Age (years)					
≤75 (319)	19.8 (17.0–21.7)				
>75 (126)	16.9 (14.1–21.0)	1.00 (0.79–1.27)	.979	–	–
Sex					
Male (238)	18.1 (15.8–20.5)	1.10 (0.89–1.36)	.382	–	–
Female (207)	20.2 (16.9–23.2)				
Tumor location					
Head (182)	18.1 (15.7–23.2)	1.04 (0.83–1.28)	.750	–	–
Body‐tail (254)	19.6 (16.7–21.7)				
Entire (9)	14.6 (3.7–22.0)	1.33 (0.62–2.82)	.463		
Resectability classification					
Resectable (353)	21.0 (18.2–23.2)			–	–
Borderline resectable (92)	14.3 (11.9–16.3)	1.59 (1.23–2.05)	<.001	1.43 (1.07–1.91)	.015
Pretreatment CA19‐9 level					
≤100 U/mL (143)	21.0 (17.1–27.8)				
>100 U/mL (246)	18.2 (15.9–21.0)	1.24 (0.98–1.57)	.072	–	–
Cytology class					
Class IV (61)	22.5 (15.5–38.4)				
Class V (384)	18.1 (16.4–21.0)	1.23 (0.91–1.65)	.180	–	–
Timing of PPC diagnosis					
After NAT (112)	19.5 (16.2–23.2)	1.12 (0.88–1.42)	.361		
Before NAT (333)	19.0 (16.5–21.2)				
Operative procedure					
PD (176)	17.7 (14.6–20.2)	1.14 (0.91–1.42)	.250	–	–
DP (243)	21.0 (17.8–23.1)				
TP (26)	13.9 (8.0–24.6)	1.31 (0.82–2.10)	.258		
Portal vein resection					
Yes (114)	15.7 (12.9–21.0)	1.29 (1.02–1.64)	.033	1.19 (0.91–1.57)	.202
No (331)	19.8 (17.7–21.9)				
Artery resection					
Yes (25)	16.7 (10.4–22.5)	1.32 (0.86–2.01)	.202	–	–
No (420)	19.0 (17.0–21.2)				
Tumor size					
≤20 mm (47)	27.9 (20.5–43.4)				
>20 mm (395)	18.1 (16.3–20.2)	1.57 (1.08–2.29)	.018	1.29 (0.85–1.98)	.233
Anterior serosal invasion					
Yes (341)	17.8 (16.3–20.2)	1.29 (0.99–1.66)	.053	–	–
No (103)	23.2 (16.2–35.2)				
Retroperitoneal invasion					
Yes (390)	18.2 (16.5–20.8)	1.52 (1.07–2.16)	.018	1.32 (0.89–1.95)	.170
No (55)	31.9 (15.9–38.4)				
Portal vein invasion					
Yes (163)	19.5 (15.9–22.0)	1.12 (0.90–1.39)	.302	–	–
No (281)	19.0 (16.5–21.4)				
Artery invasion					
Yes (52)	14.2 (9.7–21.4)	1.25 (0.91–1.72)	.174	–	–
No (392)	19.2 (17.0–21.3)				
Pathological T stage classification					
T3/T4 (253)	18.4 (16.5–21.0)	1.20 (0.97–1.49)	.093		
Tis/T1/T2 (190)	19.3 (15.9–25.9)				
Pathological lymph node metastasis					
N1/N2 (331)	19.2 (16.9–21.3)	1.01 (0.80–1.29)	.912	–	–
N0 (114)	17.1 (15.3–22.2)				
Residual cancer					
R1 (97)	17.7 (14.6–21.2)	1.18 (0.92–1.52)	.193	–	–
R0 (348)	19.8 (17.0–21.4)				
Postoperative CA19‐9 level					
≤37.0 U/mL (232)	27.6 (23.1–30.3)				
>37.0 U/mL (169)	12.2 (10.5–15.4)	2.30 (1.84–2.89)	<.001	2.04 (1.61–2.58)	<.001
Adjuvant treatment					
No (101)	8.8 (7.7–10.7)	2.22 (1.74–2.84)	<.001	2.16 (1.63–2.84)	<.001
Yes (343)	21.9 (19.6–25.4)				

Abbreviations: CA19‐19, carbohydrate antigen 19–9; DP, distal pancreatectomy; HR, hazard ratio; NAT, neoadjuvant treatment; OS, overall survival; PaCa, pancreatic cancer; PD, pancreaticoduodenectomy; PPC, positive peritoneal cytology; TP, total pancreatectomy.

### 3.8 Effect of adjuvant treatment in the S‐group

Adjuvant treatments were performed for 343 patients in the S‐group, among whom S‐1 was the most commonly used adjuvant treatment (283 patients, 63.6%). The MST after S‐1 treatment was 23.2 months, which was significantly better than that after treatment with gemcitabine (GEM) (16.8 months, *p* = .036), GnP (19.0 months, *p* = .030), or no adjuvant treatment (8.8 months, *p* < .001) (Figure S6). With respect to the duration of adjuvant treatment, 132 (29.7%) patients received treatment beyond the commonly recommended 6‐month period.^19^ The MST of patients who underwent >6 months of adjuvant treatment was significantly better (MST, 30.3 months) than that of patients who received adjuvant treatment for 3–6 months (MST, 21.4 months; *p* = .005), 1–3 months (MST, 12.0; *p* < .001), or <1 month (MST, 7.8; *p* < .001) (Figure S7). As favorable adjuvant treatment, if S‐1 was used for >6 months, the MST of the corresponding 104 patients could reach 34.9 months (data not shown).

## 4 DISCUSSION

According to several clinical guidelines, surgical resection is not recommended for patients with PaCa having PPC. However, postoperative survival for patients with PPC is not as unfavorable and retains the possibility of curability via surgical resection, unlike other M1 diseases.^10^, ^20^ Therefore, PPC has not been strictly recognized as a contraindication to pancreatectomy, and studies aiming to improve postoperative prognosis have been reported.^21^ These studies highlight the need for careful consideration of complete rejection for surgery and suggest the necessity to further clarify the optimal treatment for PPC. To address this issue, this study elucidated the prognosis of both surgical and nonsurgical treatment for PPC. The study findings indicate that surgical resection is not beneficial for improving OS in patients with PaCa when PPC is evident. In the N‐group, continuation with only nonsurgical treatment would be insufficient, given the findings compared with those in the S‐group. In contrast, CS for appropriately selected patients markedly prolonged survival. These results suggest that preceding chemotherapy aiming to achieve CS is desirable for patients with PPC.

The prognosis of patients who achieved CS was favorable even after resection. This may be due to the effects of systemic chemotherapy before achieving CS. In the S‐group, more than 40% of the patients did not achieve CA19‐9 normalization after resection, and postoperative recurrence frequently occurred in distant areas, such as the peritoneum or liver. This recurrence may result from microresidual cancer following resection with PPC.^22^ In contrast, when CS could be performed, over 90% of the patients achieved postoperative CA19‐9 normalization, and distant recurrence was drastically suppressed. This implies that chemotherapy until CS may contribute to the disappearance of potential micrometastases. Taken together, these findings indicate that PPC should be treated as a systemic disease, and nonsurgical treatment with strong chemotherapy may be desirable as initial treatment. To achieve a favorable outcome, aiming for CS after identifying appropriate patients is crucial.

The CS rate of 24.6% in our study was relatively low compared with previously reported rates of 31.8%–52.2%.^14^, ^23^ The incidence of CS depends on the treatment strategy; thus, an appropriate method to achieve CS must be identified. In this study, two predictive factors contributing to CS were identified: the timing of diagnosis and the regimens of initial treatment. When PPC was diagnosed after NAT, the proportion of patients who achieved CS was low (approximately 5%). PPC after NAT might be interpreted in the following two ways: persistence of PPC before NAT or a new appearance during NAT. In addition, patients whose cytology status changed from positive to negative were not included in this cohort. Therefore, the difficulty of CS after NAT might have resulted from poorer sensitivity to chemotherapy. The treatment regimen is another important factor. Combination treatment involving GnP or FOLFIRINOX is usually used for unresectable or recurrent PaCa^24^, ^25^ and differs from the regimens typically used for resectable PaCa as NAT.^26^, ^27^ When these regimens were not used as initial treatments, the rate of CS was only 2.6%. Considering these two factors and to avoid unnecessary chemotherapy resistance owing to insufficient treatment, the evaluation of PPC before NAT and the initiation of combination treatment are currently considered appropriate for achieving CS. When combination treatment was performed before NAT, the rate of CS in the corresponding 58 patients was 46.6% in this study (data not shown).

Determining adequate criteria for CS is important. In this study, CS was performed in patients who achieved a negative conversion of cytological status and had no other evident unresectable factors. These criteria are virtually equivalent to previously suggested criteria,^28^ and may be good indicators for deciding on CS when a favorable prognosis following CS is considered. One major issue remaining to be resolved is the optimal duration of treatment prior to CS. The median treatment period was 6.0 months in this study. This duration is relatively short compared with the previously suggested 8 months prior to CS for initially unresectable pancreatic cancer.^29^ This study did not evaluate the prognostic effect of treatment duration; therefore, whether 6 months of treatment is sufficient for CS remains to be determined. Additional studies are needed to determine the optimal duration of treatment considering the balance between the rate of achieving CS and the prognosis after CS.

The results of the study questionnaire revealed that approximately 85% of the surveyed Japanese institutions conducted intraoperative diagnoses to evaluate peritoneal washing cytology. The intraoperative diagnosis is uncertain and may change from negative to positive upon final diagnosis. Therefore, elucidating adequate treatment after surgery with PPC is helpful in the clinical setting. In this study, adjuvant treatment was identified as a potential intervention factor contributing to patient prognosis. Further analysis of treatment regimens indicated that the prognosis was better when S‐1 monotherapy was used than when GEM or GnPs were used. Although determining the effect of GnPs is challenging owing to the small number of patients, S‐1 might be preferable as an adjuvant treatment. The duration of adjuvant treatment is favorable if it continues to exceed the commonly recommended 6 months, as reported by Ohgi et al.^22^ The results concerning the effect of long‐term adjuvant treatment may have involved selection bias because this cohort excluded patients who could not continue chemotherapy owing to side‐effects or recurrence. Nevertheless, long‐term adjuvant treatment can be considered effective because it appears to increase the likelihood of achieving remission after surgical resection. Taken together, our findings suggest that the optimal treatment for PPC after resection may be long‐term adjuvant chemotherapy with S‐1. However, even when favorable adjuvant treatment was successfully administered in the S‐group, the prognosis did not exceed that of patients who achieved CS in the N‐group.

In summary, the findings of this study indicate that surgical resection is not superior to nonsurgical treatment for patients with PaCa having PPC. This implies that surgery at the time of PPC cannot provide a benefit commensurate with invasiveness. In contrast, according to the results of predictive factors for survival in the S‐ and N‐groups, chemotherapy is a key treatment for improving prognosis. In the past, few effective systemic chemotherapeutic regimens were available. Therefore, past studies advocated the significance of surgical resection, even in patients with PPC, and CS was rarely performed. Recent developments in chemotherapy have led to a paradigm shift wherein the significance of surgical resection has diminished and chemotherapy is the preferred treatment for PPC. However, continuation of chemotherapy alone appears to be insufficient for improving survival. Although CS is performed for selected patients with good sensitivity to chemotherapy, this is a powerful factor that improved survival in the N‐group. Therefore, the goal of PPC treatment should be to achieve CS, and maximizing the effects of chemotherapy might be preferable as an initial treatment.

This retrospective study had several limitations. First, PPC treatment was performed according to the policies of each center. Therefore, PPC treatment regimens or clear criteria for CS were not determined. However, certain standards are assured for CS, as it is performed only for patients with negative cytology and no other progressive disease. Second, a central review of the cytological status was not performed. The determination of Class IV or V depended on the diagnosis at each center, and there may have been minor differences in the PPC diagnoses according to the center. Third, this study collected data from JSHBPS board‐certified institutions.^15^ Thus, the data may not necessarily be representative of the entire country and may have introduced institutional bias. Finally, propensity score matching was used in the analysis; however, residual confounding factors could not be addressed using this evaluation method alone. Nevertheless, this is the largest reported study to suggest that surgical resection is not superior to nonsurgical treatment in patients with PaCa having PPC. Although the prognosis of nonsurgical treatment might have depended on the powerful factor as CS, the results of this study are likely to help resolve issues concerning indications for surgical resection of PPC. Recently, staging laparoscopy has become widespread for pancreatic cancer, and PPC has been more commonly detected than it was previously.^23^, ^30^ Because of the increasing number of patients with PPC, treatment strategies for PPC need to be rapidly confirmed. These study results provide support for initial chemotherapy for PPC with CS in selected patients. While this study proposes an optimal treatment strategy for PPC, several issues remain unresolved. Future studies are needed to clarify the appropriate treatment regimens and criteria for CS to establish more effective and desirable treatment strategies for patients with PaCa having PPC.

## 5 CONCLUSION

Surgical resection at the time of PPC may not improve OS compared with nonsurgical treatment. Given that CS can be expected for approximately 25% patients and markedly improves the prognosis, initiation of chemotherapy aimed at CS is optimal for patients with PPC. In future studies, the appropriate treatment for achieving CS should be clarified considering the optimal treatment regimens and timing of resection.

## CONFLICT OF INTEREST STATEMENT

The authors declare no conflict of interest for this article.

## Supporting information


Figures S1–S7

Tables S1–S3
