# Supplementary material for: Optimal treatment strategy for patients with pancreatic cancer having positive peritoneal cytology: A nationwide multicenter retrospective cohort study supervised by the Japanese Society of Hepato‐Biliary‐Pancreatic Surgery
Source: J Hepatobiliary Pancreat Sci. 2024 Sep 24;32(1):69–81. doi: 10.1002/jhbp.12074 (PMC11780303; doi:10.1002/jhbp.12074)
Supplement: Supplementary file 1 — Figures S1–S7 [file JHBP-32-69-s001.pptx]

## Slide 1
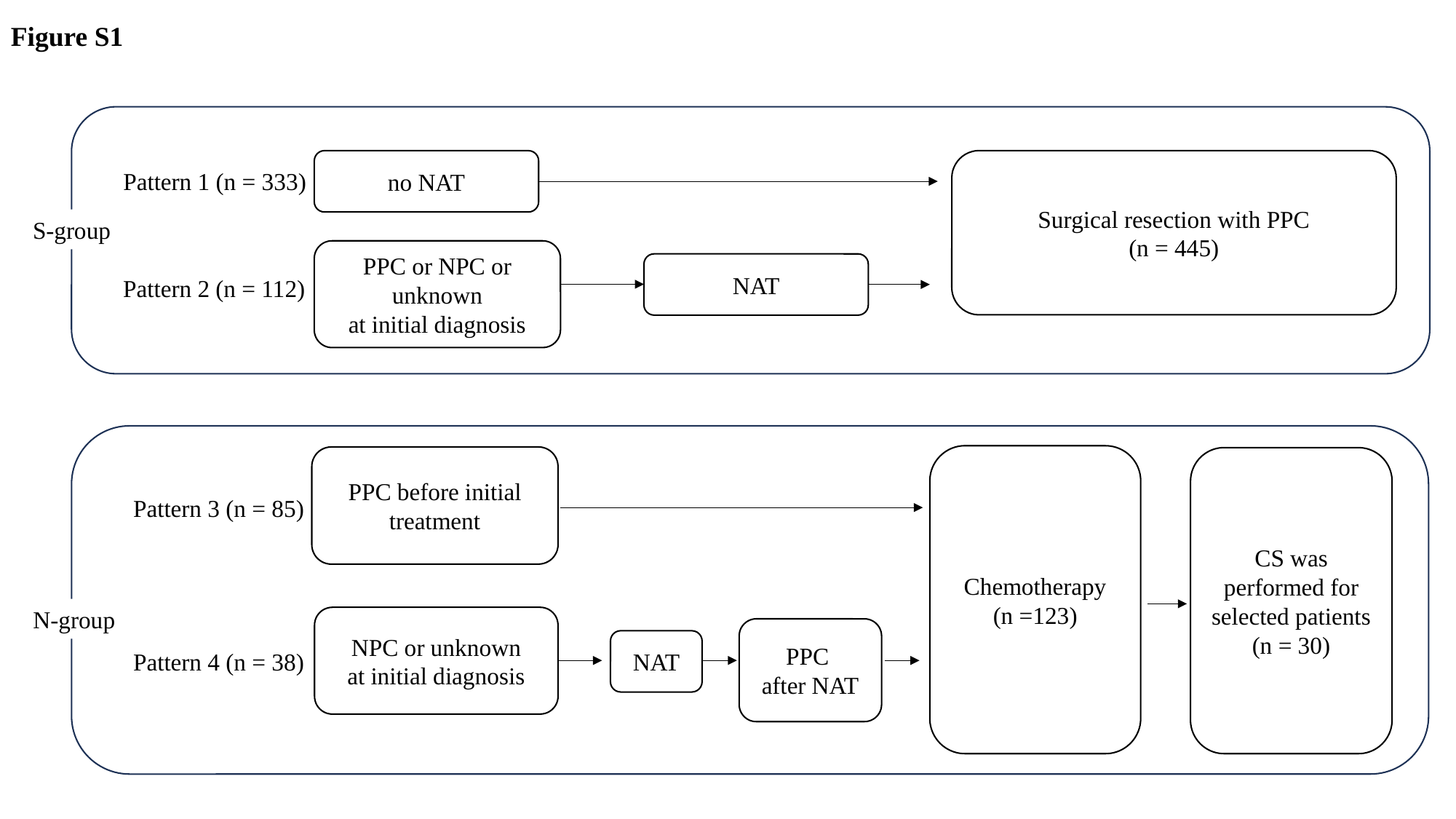

Figure S1
no NAT
Surgical resection with PPC
(n = 445)
Pattern 1 (n = 333)
S-group
PPC or NPC or unknown
at initial diagnosis
NAT
Pattern 2 (n = 112)
Chemotherapy
(n =123)
PPC before initial treatment
CS was performed for selected patients
(n = 30)
Pattern 3 (n = 85)
N-group
NPC or unknown
at initial diagnosis
PPC
after NAT
NAT
Pattern 4 (n = 38)

## Slide 2
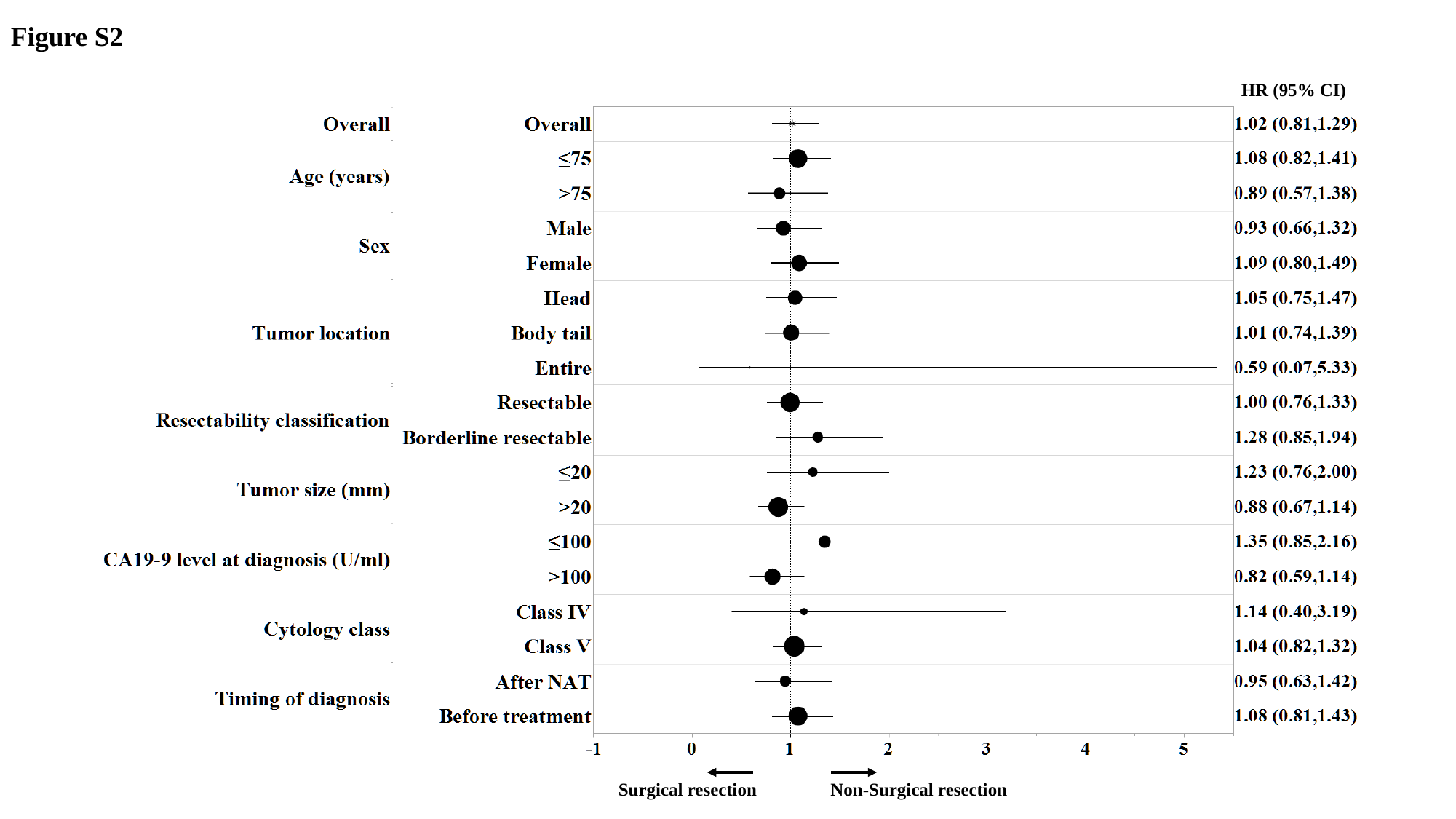

Figure S2
HR (95% CI)
Surgical resection
Non-Surgical resection

## Slide 3
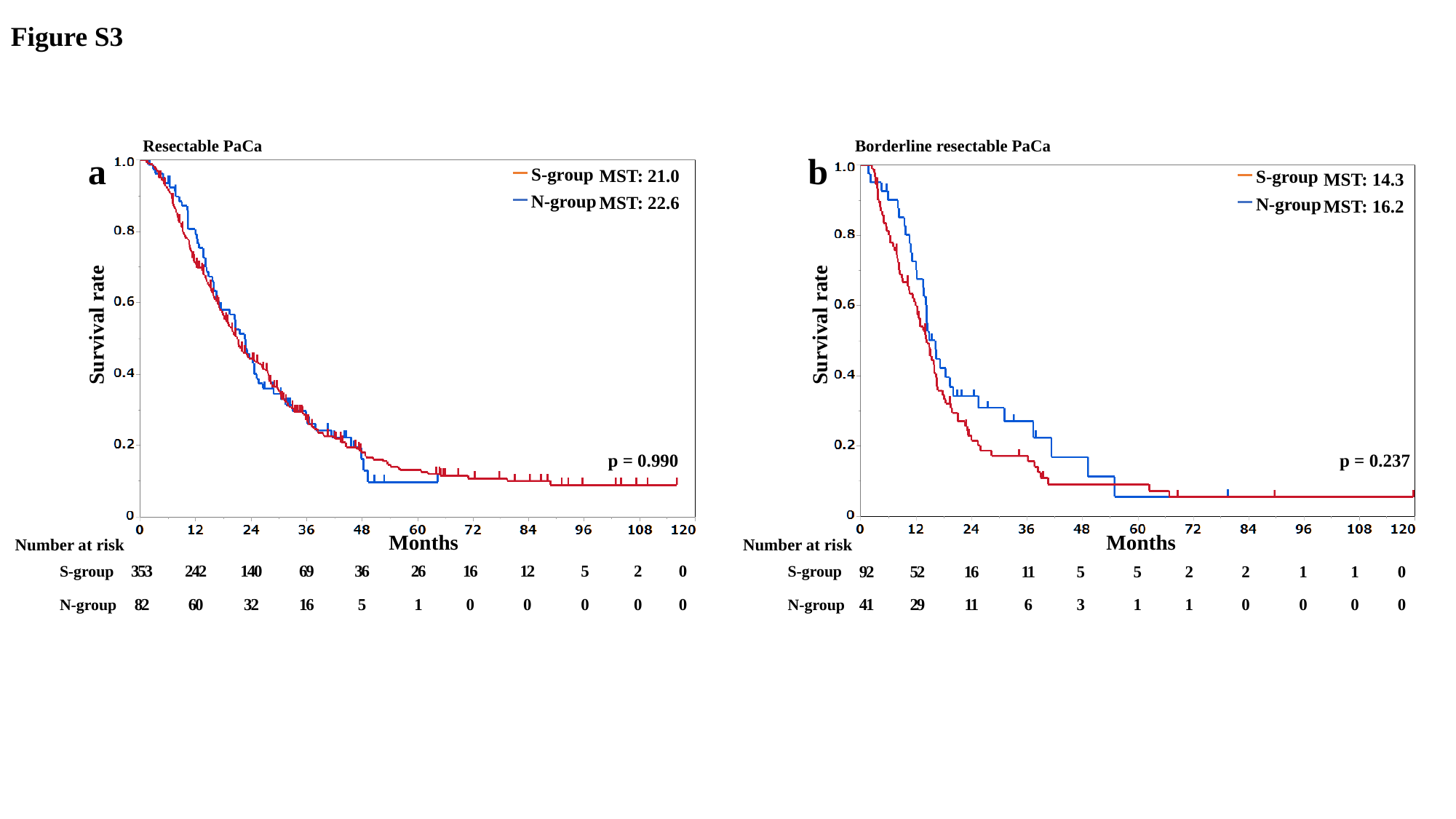

Figure S3
Resectable PaCa
Borderline resectable PaCa
a
b
S-group
N-group
MST: 21.0
S-group
N-group
MST: 14.3
MST: 22.6
MST: 16.2
Survival rate
Survival rate
p = 0.990
p = 0.237
Months
Months
Number at risk
S-group
N-group
Number at risk
S-group
N-group
| | 353 | 242 | 140 | 69 | 36 | 26 | 16 | 12 | 5 | 2 | 0 |
| --- | --- | --- | --- | --- | --- | --- | --- | --- | --- | --- | --- |
| | 82 | 60 | 32 | 16 | 5 | 1 | 0 | 0 | 0 | 0 | 0 |
| | 92 | 52 | 16 | 11 | 5 | 5 | 2 | 2 | 1 | 1 | 0 |
| --- | --- | --- | --- | --- | --- | --- | --- | --- | --- | --- | --- |
| | 41 | 29 | 11 | 6 | 3 | 1 | 1 | 0 | 0 | 0 | 0 |

## Slide 4
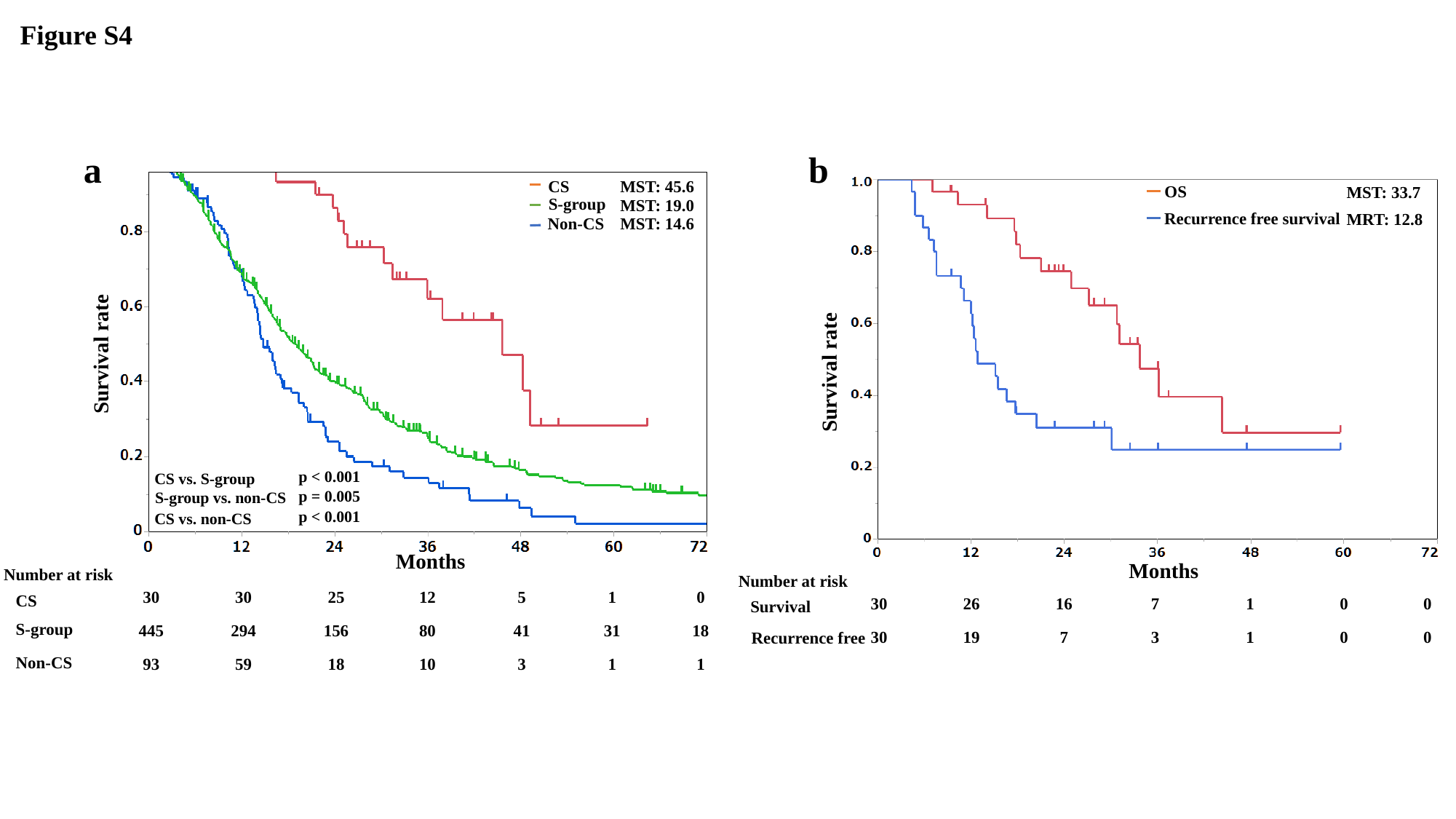

Figure S4
a
b
CS
MST: 45.6
S-group
MST: 19.0
MST: 14.6
Non-CS
OS
Recurrence free survival
MST: 33.7
MRT: 12.8
Survival rate
Survival rate
p < 0.001
CS vs. S-group
p = 0.005
S-group vs. non-CS
p < 0.001
CS vs. non-CS
Months
Months
Number at risk
Number at risk
| | 30 | | 30 | | 25 | | 12 | | 5 | | 1 | | 0 | |
| --- | --- | --- | --- | --- | --- | --- | --- | --- | --- | --- | --- | --- | --- | --- |
| | 445 | | 294 | | 156 | | 80 | | 41 | | 31 | | 18 | |
| | 93 | | 59 | | 18 | | 10 | | 3 | | 1 | | 1 | |
CS
| | 30 | | 26 | | 16 | | 7 | | 1 | | 0 | | 0 | |
| --- | --- | --- | --- | --- | --- | --- | --- | --- | --- | --- | --- | --- | --- | --- |
| | 30 | | 19 | | 7 | | 3 | | 1 | | 0 | | 0 | |
Survival
Recurrence free
S-group
Non-CS

## Slide 5
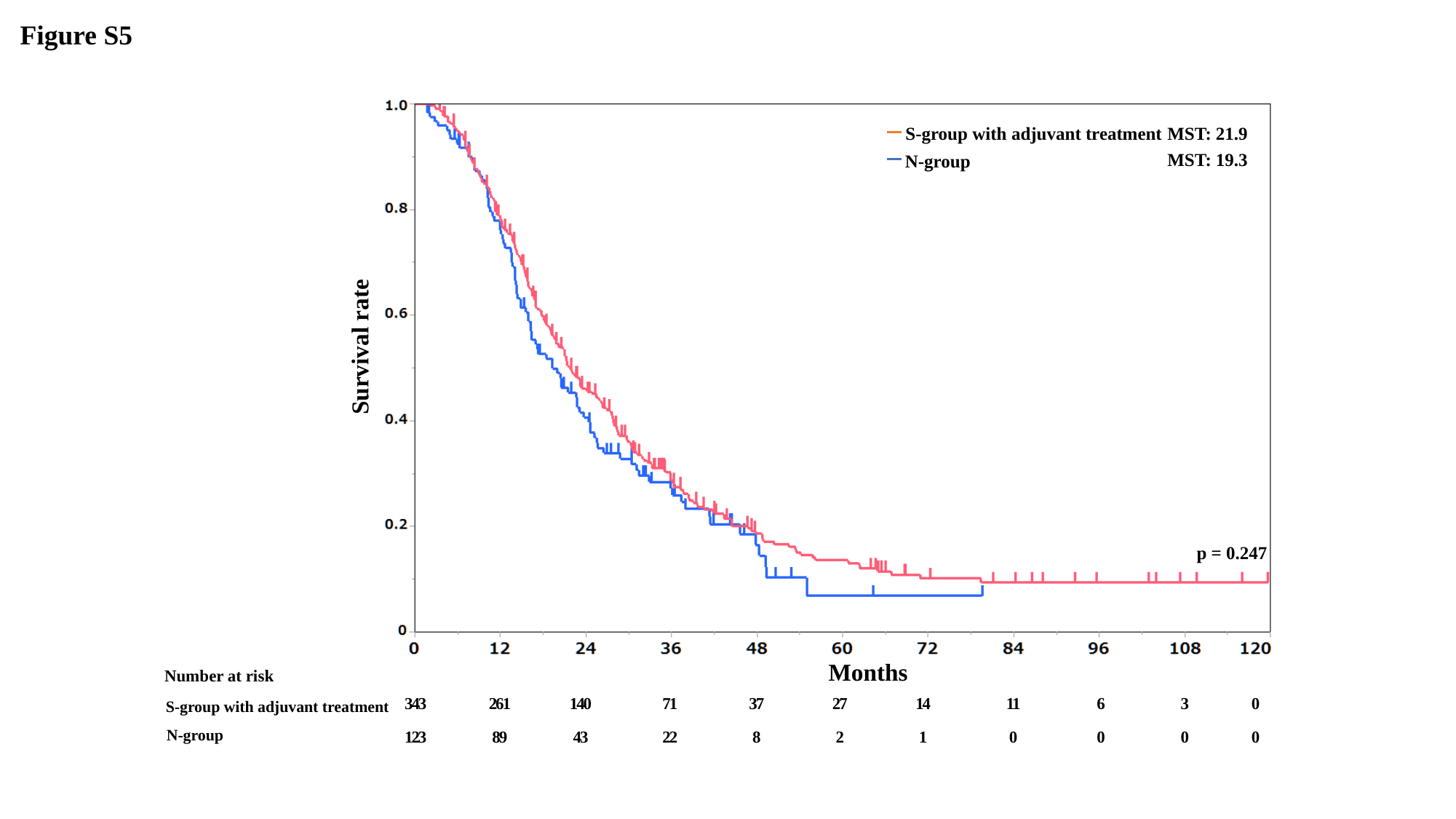

Figure S5
MST: 21.9
S-group with adjuvant treatment
N-group
MST: 19.3
Survival rate
p = 0.247
Months
Number at risk
S-group with adjuvant treatment
N-group
| | 343 | 261 | 140 | 71 | 37 | 27 | 14 | 11 | 6 | 3 | 0 |
| --- | --- | --- | --- | --- | --- | --- | --- | --- | --- | --- | --- |
| | 123 | 89 | 43 | 22 | 8 | 2 | 1 | 0 | 0 | 0 | 0 |

## Slide 6
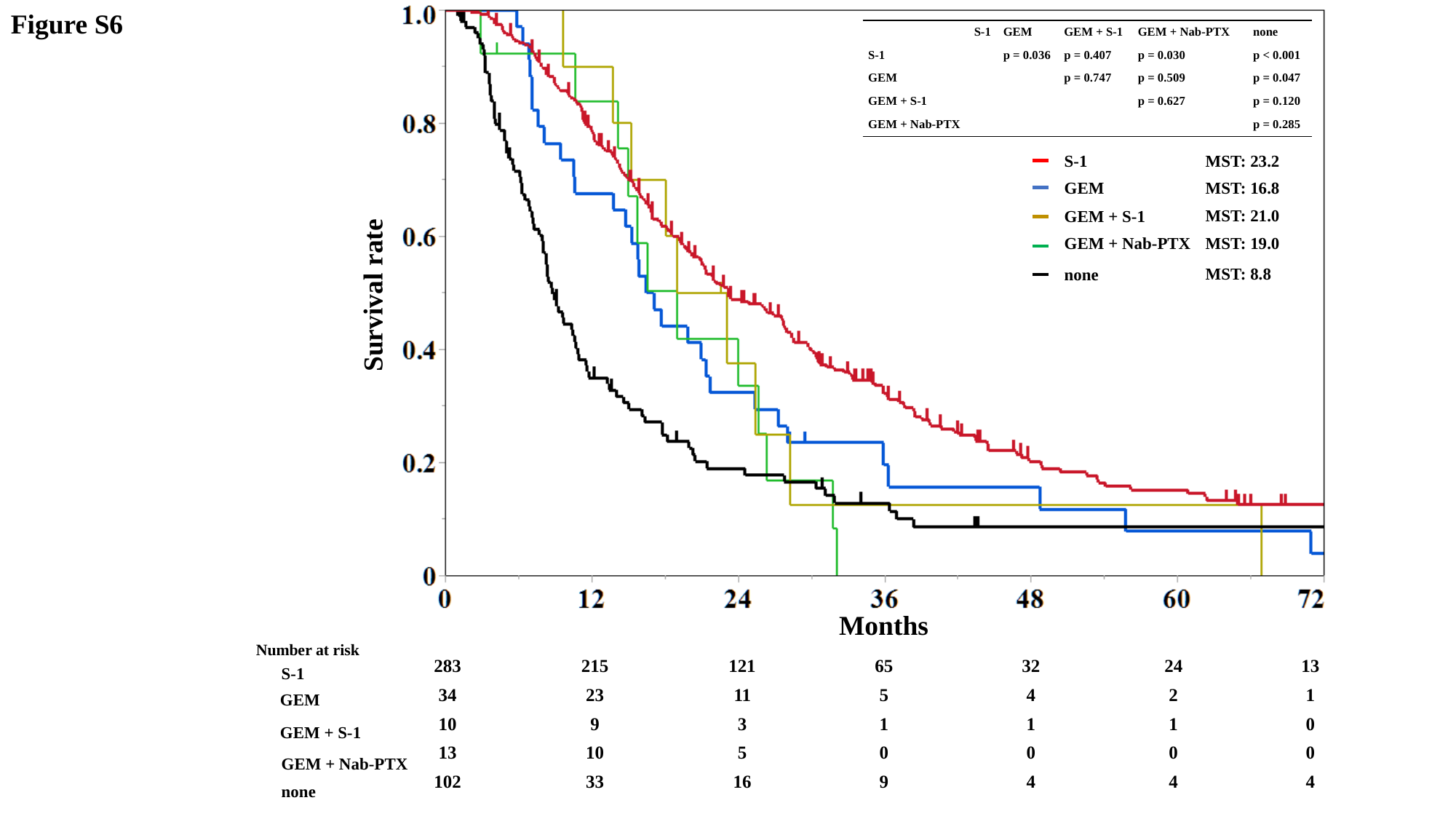

Figure S6
| | S-1 | GEM | GEM + S-1 | GEM + Nab-PTX | none |
| --- | --- | --- | --- | --- | --- |
| S-1 | | p = 0.036 | p = 0.407 | p = 0.030 | p < 0.001 |
| GEM | | | p = 0.747 | p = 0.509 | p = 0.047 |
| GEM + S-1 | | | | p = 0.627 | p = 0.120 |
| GEM + Nab-PTX | | | | | p = 0.285 |
S-1
GEM
GEM + S-1
GEM + Nab-PTX
none
MST: 23.2
MST: 16.8
MST: 21.0
MST: 19.0
MST: 8.8
Survival rate
Months
Number at risk
| | 283 | | 215 | | 121 | | 65 | | 32 | | 24 | | 13 | |
| --- | --- | --- | --- | --- | --- | --- | --- | --- | --- | --- | --- | --- | --- | --- |
| | 34 | | 23 | | 11 | | 5 | | 4 | | 2 | | 1 | |
| | 10 | | 9 | | 3 | | 1 | | 1 | | 1 | | 0 | |
| | 13 | | 10 | | 5 | | 0 | | 0 | | 0 | | 0 | |
| | 102 | | 33 | | 16 | | 9 | | 4 | | 4 | | 4 | |
S-1
GEM
GEM + S-1
GEM + Nab-PTX
none

## Slide 7
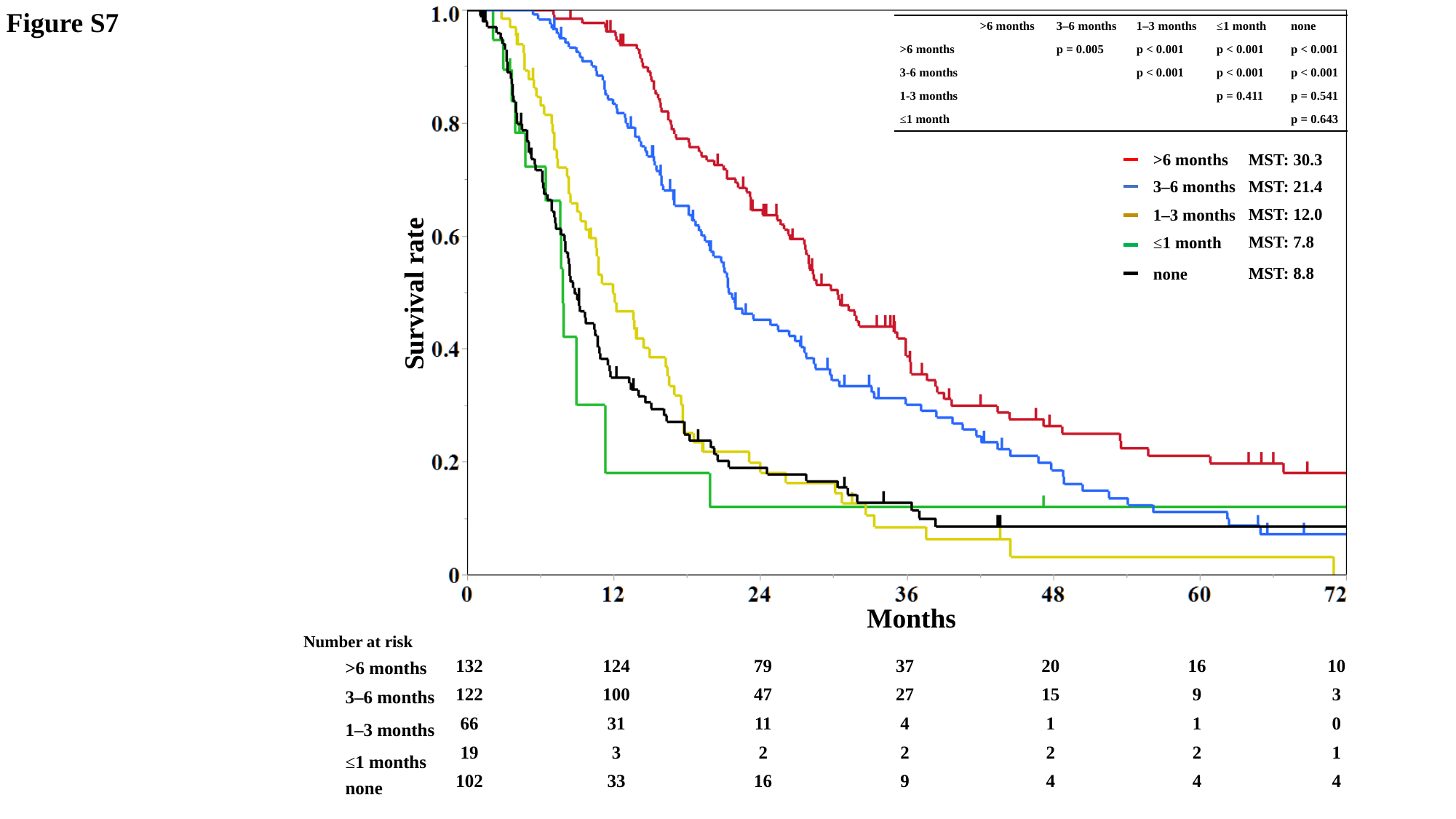

Figure S7
| | >6 months | 3–6 months | 1–3 months | ≤1 month | none |
| --- | --- | --- | --- | --- | --- |
| >6 months | | p = 0.005 | p < 0.001 | p < 0.001 | p < 0.001 |
| 3-6 months | | | p < 0.001 | p < 0.001 | p < 0.001 |
| 1-3 months | | | | p = 0.411 | p = 0.541 |
| ≤1 month | | | | | p = 0.643 |
>6 months
3–6 months
1–3 months
≤1 month
none
MST: 30.3
MST: 21.4
MST: 12.0
MST: 7.8
MST: 8.8
Survival rate
Months
Number at risk
>6 months
| | 132 | | 124 | | 79 | | 37 | | 20 | | 16 | | 10 | |
| --- | --- | --- | --- | --- | --- | --- | --- | --- | --- | --- | --- | --- | --- | --- |
| | 122 | | 100 | | 47 | | 27 | | 15 | | 9 | | 3 | |
| | 66 | | 31 | | 11 | | 4 | | 1 | | 1 | | 0 | |
| | 19 | | 3 | | 2 | | 2 | | 2 | | 2 | | 1 | |
| | 102 | | 33 | | 16 | | 9 | | 4 | | 4 | | 4 | |
3–6 months
1–3 months
≤1 months
none
