# Supplementary material for: Optimal treatment strategy for patients with pancreatic cancer having positive peritoneal cytology: A nationwide multicenter retrospective cohort study supervised by the Japanese Society of Hepato‐Biliary‐Pancreatic Surgery
Source: J Hepatobiliary Pancreat Sci. 2024 Sep 24;32(1):69–81. doi: 10.1002/jhbp.12074 (PMC11780303; doi:10.1002/jhbp.12074)
Supplement: Supplementary file 2 — Tables S1–S3 [file JHBP-32-69-s002.docx]

**Supplemental Tables**

**Table S1.** Participating institutions

| Shimane University Faculty of Medicine |
| --- |
| Kagoshima University |
| Kagoshima City Hospital, Kagoshima |
| Kitano Hospital Medical Research Institute |
| Hyogo Cancer Center |
| Hokkaido University Faculty of Medicine |
| Ibaraki Medical Center, Tokyo Medical University |
| Fujita Health University Bantane Hospital |
| Kumamoto University |
| Higashiosaka City Medical Center |
| Hirosaki University Graduate School of Medicine |
| Hyogo College of Medicine |
| Iwata City Hospital |
| Kagawa University School of Medicine |
| Nagoya University Graduate School of Medicine |
| Niigata Cancer Center Hospital |
| Osaka University Graduate School of Medicine |
| Sendai City Medical Center Sendai Open Hospital |
| Sendai Kousei Hospital |
| Yokohama City University Graduate School of Medicine |
| Yamaguchi University Graduate School of Medicine |
| Japanese Red Cross Osaka Hospital |
| Nippon Medical School |
| Tokyo Medical University |
| Ehime University Graduate School of Medicine |
| Kanagawa Cancer Center |
| Kitasato University Hospital |
| Gunma University |
| National Cancer Center Hospital East |
| Mie University Graduate School of Medicine |
| Fukushima Medical University |
| Kyorin University Faculty of Medicine |
| Kyushu University |
| University of Toyama |
| National Hospital Organization Sendai Medical Center |
| Ageo Central General Hospital |
| Chibanishi General Hospital |
| Ehime Prefectural Central Hospital |
| Saga University |
| Kyoto University |
| Hokkaido P.W.F.A.C Sapporo Kosei General Hospital |
| Iizuka Hospital |
| Tokyo Women’s Medical University |
| Iwaki City Medical Center |
| JA Hiroshima General Hospital |
| Japanese Red Cross Ishinomaki Hospital |
| Kansai Medical University |
| Kawasaki Municipal Hospital |
| Kindai University Faculty of Medicine |
| Kitakyushu Municipal Medical Center |
| Kobe City Medical Center General Hospital |
| Konan Medical Center |
| Kurume University School of Medicine |
| Mito Kyodo General Hospital |
| Nagaoka Chuo General Hospital |
| National Hospital Organization Nagoya Medical Center |
| National Hospital Organization, Kure Medical Center and Chugoku Cancer Center |
| Niigata Prefectural Central Hospital |
| Osaka International Cancer Institute |
| Osaka Saiseikai Nakatsu Hospital |
| Otsu Red Cross Hospital |
| Saitama City Hospital |
| Shiga General Hospital |
| Shiga University of Medical Science |
| Shinshu University school of medicine |
| Sapporo Medical University |
| Tohoku University Graduate School of Medicine |
| Toyota Kosei Hospital |
| Tsuyama Chuo Hospital |
| Gifu University Hospital |
| Niigata University |
| Tottori University Faculty of Medicine |
| Kobe University Graduate School of Medicine |
| University of Miyazaki Faculty of Medicine |
| Nagoya University Graduate School of Medicine |
| Saitama Cancer Center |
| Dokkyo Medical University |
| Wakayama Medical University School of Medicine |

**Table S2.** Current treatment strategies for patients with pancreatic cancer having positive peritoneal cytology

| **Primary survey** | |
| --- | --- |
| **Question** | **Response from 86 centers** |
| Treatment for PPC | Surgical resection (n = 52) |
|  | Non-surgical treatment (n = 32) |
|  | Surgical resection for DP, non-surgical resection for PD (n = 2) |
| **Secondary survey** | |
| **Question** | **Response from 55 centers** |
| Method of diagnosis | With intraoperative diagnosis (n = 47) |
|  | Only permanent diagnosis (n = 8) |
| Staging laparoscopy | For all patients (n = 4) |
|  | Selected patients (n = 39) |
|  | None (n = 12) |

**Abbreviations:** DP, distal pancreatectomy; PD, pancreatoduodenectomy; PPC, positive peritoneal cytology

**Table S3.** Clinical characteristics of patients who underwent conversion or non-conversion surgery

| **Factor** |  | **Conversion surgery (n = 30)** | **Non-conversion surgery (n = 93)** | **p-value** |
| --- | --- | --- | --- | --- |
| Age (years) | Median (range) | 66.5 (51–80) | 71 (35–88) | 0.097 |
| Sex | Male/female | 21/9 | 46/47 | 0.049 |
| Tumor location | Head/Body-Tail/entire | 12/18/0 | 49/43/1 | 0.382 |
| Resectability classification | Resectable /Borderline resectable | 25/5 | 57/36 | 0.026 |
| Tumor size (mm) | Median (range) | 23.5 (10–50) | 30 (0–70) | 0.104 |
| Pretreatment CA19-9 level (U/mL ) | Median (range) | 83.7 (5.2–5667.7) | 161 (2.8–15223) | 0.125 |
| Cytology class | Class Ⅳ/class Ⅴ | 2/28 | 3/90 | 0.407 |
| Timing of PPC diagnosis | After NAT/Before NAT | 2/28 | 36/57 | 0.001 |
| Treatment regimens | mFOLFIRINOX | 4 | 9 | 0.041 |
|  | GEM + Nab - PTX | 25 | 47 |  |
|  | GEM +S-1 | 1 | 8 |  |
|  | GEM monotherapy | 0 | 8 |  |
|  | S-1 monotherapy | 0 | 6 |  |
|  | CRT | 0 | 4 |  |
|  | other | 0 | 3 |  |
|  | none | 0 | 8 |  |
| Cytology status | Conversion to negative | 30 | 13 |  |
|  | Re-conversion from negative to positive | 0 | 4 |  |
|  | Remains positive | 0 | 6 |  |
|  | No examination | 0 | 70 |  |
| Treatment duration to CS (month) | Median (range) | 6.0 (1.9–17.8) | n.d |  |
| Regimen course pre-CS | 1^st^ line : 2^nd^ line | 27 : 3 | n.d |  |

**Abbreviations:** CA19-9, carbohydrate antigen 19-9; CRT, chemoradiotherapy; CS, conversion surgery; GEM, gemcitabine; Nab-PTX, nab-paclitaxel; NAT, neoadjuvant treatment; n.d, no data; PPC, positive peritoneal cytology
